# Editorial for the Special Issue on Wireless Microdevices and Systems for Biomedical Applications

**DOI:** 10.3390/mi9030112

**Published:** 2018-03-05

**Authors:** Paulo Mateus Mendes

**Affiliations:** CMEMS, University of Minho, 4800-058 Guimarães, Portugal; paulo.mendes@dei.uminho.pt; Tel.: +351-253-510190

Wireless microdevices are getting smaller and smaller, and in this special issue seven papers address a few miniaturization challenges in the biomedical field, which are common across different applications. Kargaran et al. [1] proposes a new ultra-low-voltage ultra-low-power LNA, where the reduced current consumption of only 160 μA, on a supply as low as 0.18 V, has potential to enable future RF receivers for ultra-low-power implantable devices. Lo et al. [2] presents an implantable functional electrical stimulation (IFES) for gastrointestinal (GI) tract modulation to treat GI dysmotility. They present a miniaturized wireless implant capable of modulating and recording GI motility. The integration challenge was addressed, where the implant incorporates a custom-made system-on-a-chip (SoC), and a heterogeneous system-in-a-package (SiP) to achieve device miniaturization and integration. The developed technology was used for in vivo experiments using both rodent and porcine models to validate the effectiveness of the implant. Zhang et al. [3] also addressed the integration challenge of device miniaturization, while keeping in mind that such systems need to be connected to external devices, e.g., to develop closed loop control devices. They present the design of a wireless, implantable, multi-channel, programmable stimulator with arbitrary channel combination, which is able to establish a wireless communication link between a specified Android-based graphical user interface (GUI). The experimental results demonstrated a successful independent configuration between different channels, as well as an arbitrary channel combination of the implantable device. Li et al. [4] presented a device that features both wireless power and communications using the same pair of coils. The developed device allows a transcorneal stimulation with an amplitude range of 5 μA to 320 μA, a frequency range of 10 Hz to 160 Hz, and a duty ratio range of 2.5% to 20%. After coating with biocompatible silicone, the whole implant has dimensions only of 6 mm in diameter, with a thickness of less than 1 mm. The full system functionality and electrical performance were demonstrated with measurement results. The work by Kim et al. [5] is related with the propulsion of a mm-sized robot. In their work, after design and system optimization, a 3-mm micro-robot that simultaneously generated a propulsion force and torque, while receiving electrical energy via wireless power transfer, was successfully fabricated and verified. The work by Fernandes et al. [6] is related with system integration towards a full solid-state cooler with the ability to modulate the neural activity of rodents, without the use of large and unpractical water pipes. A miniaturized solid-state device was designed, featuring the potential for wireless power and communications, where it was possible to achieve neuromodulation temperatures lower than 30°C, while the hot side was kept below 43 °C. Dinis et al. [7] presented a review of representative state-of-the-art wireless power capabilities for implantable electronic devices, ranging from inductive coupling to ultrasounds. As the standard methodologies are in the limit of the power that can be safely transmitted to an implant, a new approach, capable of multiplying the available power inside a brain phantom for the same specific absorption rate (SAR) value, was proposed and verified with both simulation and measured data.
